# The normal stages of development for the California valley quail

**DOI:** 10.1371/journal.pone.0268524

**Published:** 2022-05-17

**Authors:** Shelby M. Perry, Jeffrey G. Whitt, Kelly S. Reyna

**Affiliations:** The Quail Research Laboratory, Texas A&M University-Commerce, Commerce, Texas, United States of America; Laboratoire de Biologie du Développement de Villefranche-sur-Mer, FRANCE

## Abstract

One challenge in avian embryology is establishing a standard developmental timetable, primarily because eggs incubated for identical durations can vary in developmental progress, even within the same species. For remedy, avian development is classified into distinct stages based on the formation of key morphological structures. Developmental stages exist for a few galliform species, but the literature is lacking a description of normal stages for California valley quail (*Callipepla californica*). Thus, the objective of this study was to stage and document the morphological and structural development of California valley quail. Over two laying seasons, 390 eggs were incubated at 37.8֯ C in 60% RH for ≤23 days. Eggs were opened every ≤6 hours to document embryonic development, including, blastoderm diameter, anterior angle of nostril to beak tip, and lengths of wing, tarsus, third toe, total beak, total foot, and embryo. California valley quail embryos were staged and compared to domestic chicken (*Gallus gallus domesticus*), the staging standard for galliformes, as well as Japanese quail (*Coturnix japonica*), blue-breasted quail (*Synoicus chinensis*) and northern bobwhite quail (*Colinus virginianus*). This study produced the first description of the 43 normal stages of development for California valley quail. Compared with other galliformes, the California valley quail has a different number of stages and displays developmental heterochrony in stages 1–24, and morphological and developmental differences in stages 25–hatch. The observed differences emphasize the importance of staging individual avian species instead of relying on poultry animal models or close relatives for developmental reference. This is extremely important in species-specific embryological studies that evaluate critical windows of development or evaluate the impacts of environmental change on avian development. This study also suggests that staging frequencies of ≤6 hours and egg transport protocols should be standardized for future staging studies.

## Introduction

Avian developmental embryology has been a topic of study for >5,000 years, when ancient Egyptians began to artificially incubate bird eggs and evaluate their daily progress by observing embryonic development [1]. Aristotle firmly cemented the discipline by systematically describing daily changes in the morphology and physiology of developing avian embryos [1]. Subsequent studies assessed the morphology of avian embryos to better understand embryonic development in varying environments, to compare development between species [1,2], and to contribute to the poultry and medical industry [3,4]. Daily observations of avian embryological development are fascinating but have severe limitations; mainly that eggs incubated for identical durations can vary in their degree of development, even within the same species [5,6]. Thus, when making comparisons, inter- or intra-specific, incubation time is an inaccurate measure of developmental progress. One remedy is to classify avian development into distinct, definable stages based on development, or visibility, of key morphological structures [6].

**Fig 1:**
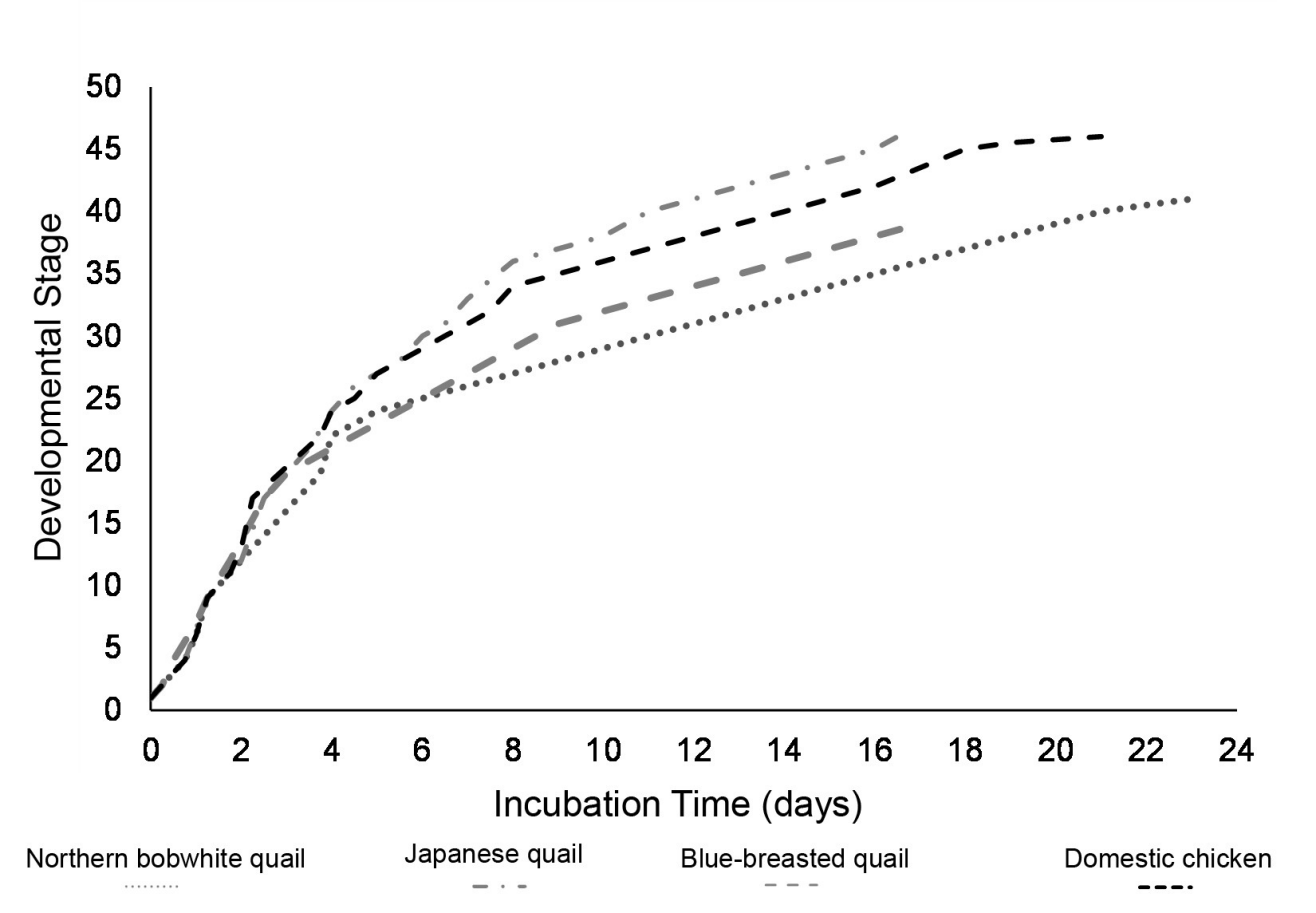
A comparison of embryo age and stages of development between northern bobwhite quail (*Colinus virginianus*), Japanese quail (*Coturnix japonica*), blue-breasted quail (*Synoicus chinensis*), and the domestic chicken (*Gallus gallus domesticus*), during incubation.

Domestic chickens (*Gallus gallus domesticus*) have historically served as the primary animal model for avian embryology. Developmental stages described for other galliform species were based on the stages of chicken embryos defined by Hamburger and Hamilton [7]. For example, early stages 1–6 were characterized by formation of the primitive streak and notochord [8]. Stages 7–14 were identified by somite formation and limb development [9–11]. Stages 15–39 were associated with external structures, including visceral arches, digit formation, and pigment development [6,12,13]. Final stages 40–46 were characterized by growth of the beak and third toe [14,15]. However, stages vary among galliforms both in rate and timing [6,7,16–18]. For example, Japanese quail (*Coturnix japonica*) embryos have 46 stages of development across 16.5 days of incubation [6], blue-breasted quail (*Synoicus chinensis*) embryos have 39 stages across 17 days [18], and northern bobwhite quail (*Colinus virginianus*) have 41 stages of development across 23 days of incubation (Fig 1) [17]. Interestingly, rates of development for bobwhite quail and chickens have no observed differences in development through stage 6 (⁓25 hours). However, bobwhites lag chicken embryos by 12–16 hours at stage 14, and ⁓24 hours by stage 24 [17]. Similarly, the gastrulation process and somite formation occur in the same order but, in the latter parts of incubation, normal stages of the chicken embryo are not applicable to other quail species.

This differential development demonstrates the need for a description of normal stages of development for each species in avian embryological studies. To date, no description of developmental stages exists for California valley quail (*Callipepla californica*). Thus, the objective of this study was to stage and document the morphological and structural development of California valley quail.

## Results

The first description of normal stages of development for the California valley quail was documented. Daily development of the California quail embryo is shown through images of opened eggs.

### Stages of development

The California valley quail has 43 normal stages of development, from pre-incubation through hatch (Table 1). Stages 1–6 were identified by primitive streak formation and gastrulation. Stages 7–14 were charted by the number of somite pairs, where every third somite pair defined a stage. Beyond stage 14, somites became increasingly more difficult to identify due to mesoderm growth on the anterior of the embryo. Other morphological structures including visceral arches, limb-buds, and external structures were identified and used to stage embryos. As development progressed, total body length, when extended, initially increased then gradually decreased at ~72 h of incubation. This was caused by cranial and cervical rotation toward the embryo’s trunk and tail region. Once rotation reached its maximum, total body length, when extended, increased for the remainder of incubation. Final stages of incubation were charted on the length of beak and third toe.

**Table 1:** Comparison of developmental stages of California quail (*Callipepla californica*), northern bobwhite quail (*Colinus virginianus*), Japanese quail (*Coturnix japonica*), blue-breasted quail (*Synoicus chinensis*), and domestic chicken (*Gallus gallus domesticus*).

Stage	California quail	Northern bobwhite [17]	Japanese quail [6]	Blue-breasted quail [18]	Domestic chicken [7,9]
1	0 h			0–9 h	
2	6–12 h			6–11 h	
3	6–12 h			10–15 h	
4	12–24 h		18–19 h	12–17 h	18–19 h
5	24–58 h		19–22 h	16–21 h	19–22 h
6	30–60 h		23–25 h	20–24 h	23–25 h
7	30–60 h		23–26 h	22–26 h	23–26 h
8	42–66 h		26–29 h	25–28 h	26–29 h
9	42–66 h		29–33 h	29–31 h	29–33 h
10	48–54 h		33–38 h	34–36 h	33–38 h
11	52–54 h		40–45 h	37–40 h	40–45 h
12	54–60 h		45–49 h	40–42 h	45–49 h
13	54 h		48–52 h	43–45 h	48–52 h
14	60 h		50–53 h	45–48 h	50–53 h
15	66 h		50–55 h	50–52 h	50–55 h
16	72–74 h		51–56 h	52–54 h	51–56 h
17	74–84 h		52–64 h	57–60 h	52–64 h
18	3.25–3.50 d		72 h	63–66 h	72 h
19	3.75 d		3 d	69–72 h	3–3.5 d
20	3.75–4 d		3.5 d	3.5 d	3–3.5 d
21	4.25–4.50 d		3.5 d	4 d	3.5 d
22	4.75–5 d		4 d	4.5 d	3.5–4 d
23	4.75–5 d		4 d	5 d	4 d
24	5 d	5 d	4 d	5.5 d	4 d
25	5.5–6 d	6 d	4.5 d	6 d	4.5 d
26	5.5–6 d	7 d	4.5–5 d	6.5 d	4.5–5 d
28	7–8 d	9 d	5.5 d	7.5 d	5.5 d
29	8–9 d	10 d	5.5–6 d	8 d	6 d
30	9 d	11 d	6–6.5 d	8.5 d	6.5 d
31	10–11 d	12 d	6.5 d	9 d	7 d
32	11–12 d	13 d	7 d	10 d	7.5 d
33	12–13 d	14 d	7 d	11 d	7.5–8 d
34	13 d	15 d	7.5 d	12 d	8 d
35	14 d	16 d	8–8.5 d	13 d	8–9 d
36	15 d	17 d	8–9 d	14 d	10 d
37	16 d	18 d	9.5 d	15 d	11 d
38	17 d	19 d	9.5–10 d	16 d	12 d
39	18 d	20 d	10.5–11 d	17 d (hatching)	13 d
40	19–20 d	21–22 d	11 d		14 d
41	20–21 d	23 d (hatching)	11.5 d		15 d
42	20–21 d		12–13 d		16 d
43	23 d (hatching)		14 d		17 d
44			15–16 d		18 d
45			16–16.5 d		19–20 d
46			16.5 d (hatching)		20–21 d (hatching)

#### Stage 1 (0 h): Pre-streak or Pre-incubation

**Fig 2:**
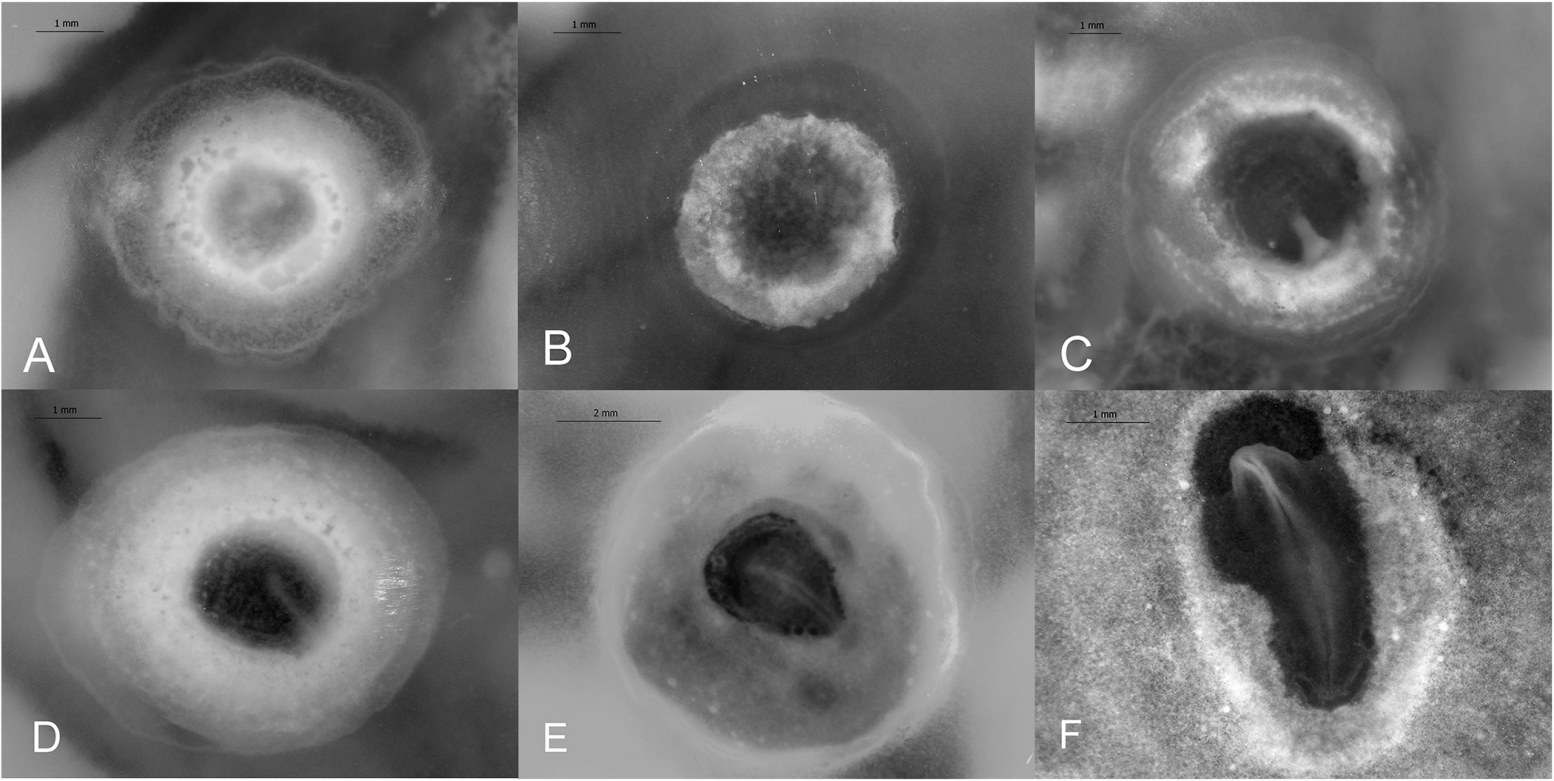
The first 60 hours (Stages 1–6) of California valley quail embryonic development. (A) Stage 1, blastoderm showing. (B) Stage 2, initial streak develops. (C) Stage 3, intermediate streak. (D) Stage 4, primitive streak with Hensen’s node. (E) Stage 5, head -process at anterior end of streak near the center of pellucida area. (F) Stage 6, head-fold, somites not yet visible.

The blastoderm’s epiblast, hypoblast, and embryonic shield are observed (N = 4; Fig 2A).

#### Stage 2 (6–12 h): Initial streak

An initial streak has formed and is 0.85 ± 0.22 mm (*N* = 3; Fig 2B). This is the start of primitive streak development and the gastrulation process. This is a transitional stage where the initial streak is almost as wide as long [7].

#### Stage 3 (6–12 h): Intermediate streak

The primitive streak has not yet reached the middle of the area pellucida. The primitive streak has become wider where it touches the opaque area. Primitive streak length: 0.89 ±0.30 mm (*N* = 2; Fig 2C).

#### Stage 4 (12–24 h): Definitive streak

Primitive streak reaches maximum length (1.40 ±0.13 mm, *N* = 8) and extends over three-quarters of the area pellucida. The area pellucida has become pear-shaped. Hensen’s node is present on the anterior end of the primitive streak (Fig 2D).

#### Stage 5 (24–58 h): Head-process

Notochord or head-process observed anterior to Hensen’s node. Head-fold has not yet developed. Embryo length: 2.15 ±0.23 mm (*N* = 6; Fig 2E).

#### Stage 6 (30–60 h): Head-fold

Head fold has begun to develop as the neural tube begins to form. Embryo length: 2.88 ±0.19 mm (*N* = 4; Fig 2F).

#### Stage 7 (30–60 h): One somite pair

**Fig 3:**
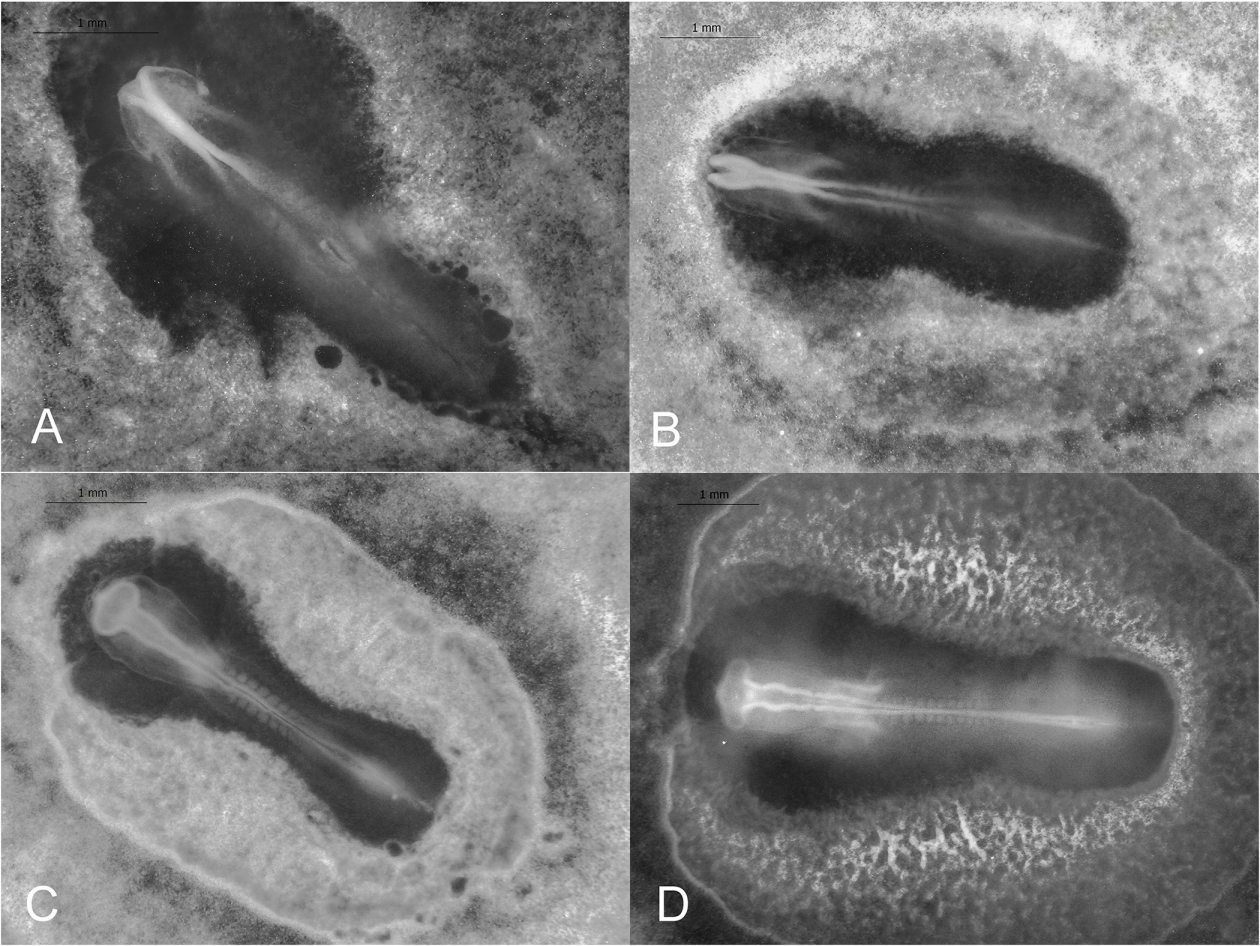
Stages 7–10 (30–54 h) of the California valley quail embryological stages of development. (A) Stage 7, one somite pair visible. (B) Stage 8, four somite pairs with blood islands present. (C) Stage 9, seven somite pairs with primordia of the heart. (D) Stage 10, 10 somite pairs and 3 brain vesicles visible.

One somite pair is visible. The neural tube begins to close to create neural groove. Embryo length: 3.61 ±0.12 mm (*N* = 5; Fig 3A).

#### Stage 8 (42–66 h): Four somite pairs

Four somite pairs are visible. Neural folds meets midbrain. Blood-islands present. Embryo length: 3.84 ±0.09 mm (*N* = 4; Fig 3B).

#### Stage 9 (42–66 h): Seven somite pairs

Seven somite pairs are visible. Primordia of heart visible. Embryo length: 4.42 ±0.15 mm (*N* = 11; Fig 3C).

#### Stage 10 (48–54 h): 10 somite pairs

Ten somite pairs visible. The first somite pair is beginning to disappear and will not be counted in subsequent stages [7,18]. Blood islands have increased. The first signs of heart and cranial rotation. Three primary brain vesicles and optic vesicles present. Embryo length: 4.90 ±0.17 mm (*N* = 8; Fig 3D).

#### Stage 11 (52–54 h): 13 somite pairs

**Fig 4:**
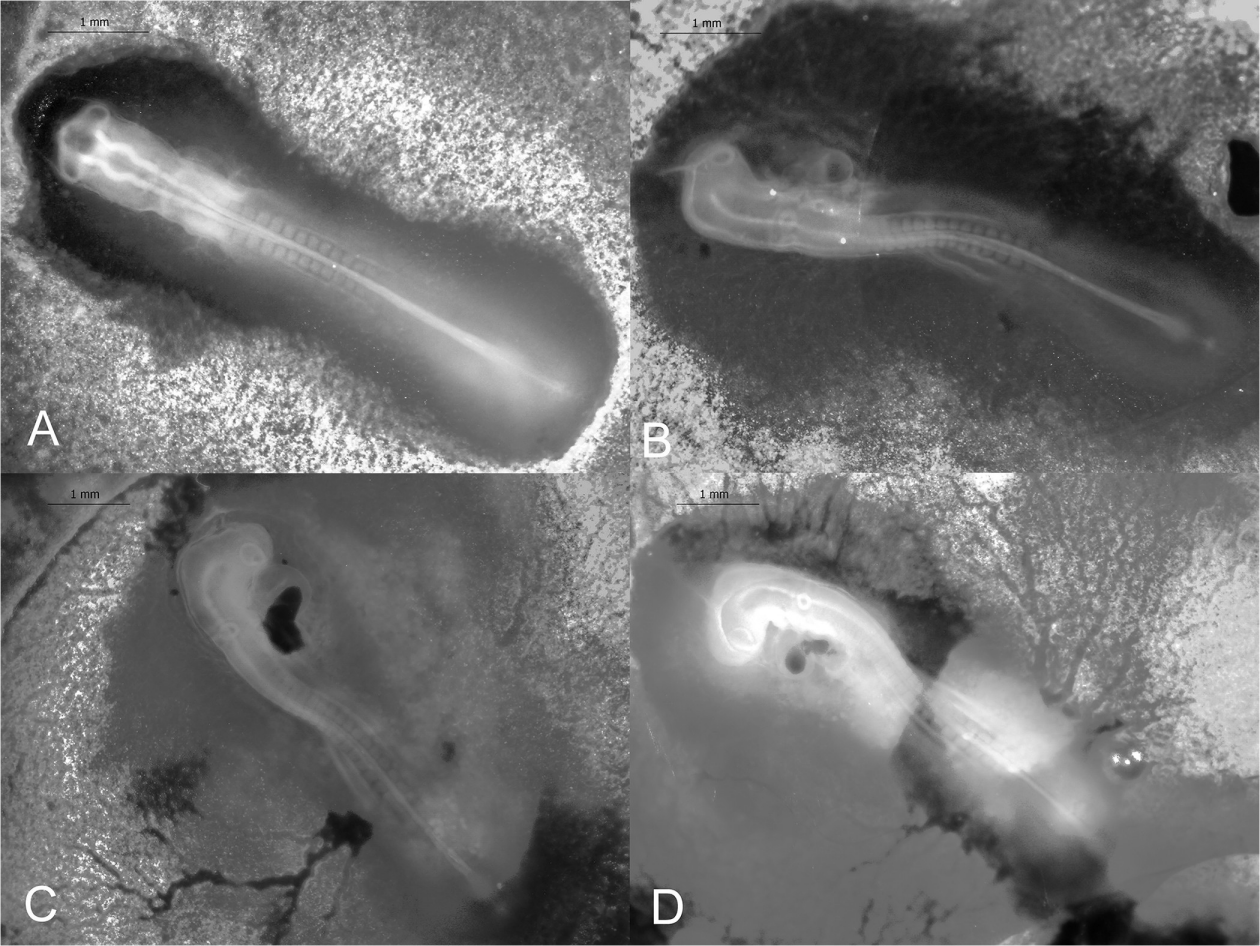
Stages 11–14 (52–60 h) of the California valley quail’s embryological stages of development. (A) Stage 11, 13 somite pairs visible. (B) Stage 12, 16 somite pairs visible. Telencephalon, optic vesicles, and auditory pit visible. (C) Stage 13, 19 somite pairs present. Telencephalon and heart more prominent than previous stage. (D) Stage 14, 22 somite pairs present. Optic lens, and Rathke’s pouch can be identified.

Thirteen somite pairs visible. The heart has started to bend to the right of the embryo. Embryo length: 5.09 ±0.54 mm (*N* = 2; Fig 4A).

#### Stage 12 (54–60 h): 16 somite pairs

Sixteen somite pairs visible. Head is turned to the left. Telencephalon, optic vesicles, and auditory pit present. Embryo length: 5.79 ±0.34 mm (*N* = 3; Fig 4B).

#### Stage 13 (54 h): 19 somite pairs

Nineteen somite pairs visible. Telencephalon is enlarged and has turned farther to the right. Heart muscle is more prominent than the previous stage (Fig 4C).

#### Stage 14 (60 h): 22 somite pairs

Twenty-two somite pairs visible. Optic lens, auditory pit, and Rathke’s pouch can be identified. Visceral arches 1 and 2 present. First and second branchial arches observed. Forebrain and hindbrain’s rotation is close to 90°. The amnion extends to somites 2–7 (Fig 4D).

#### Stage 15 (66 h): 24 somite pairs

**Fig 5:**
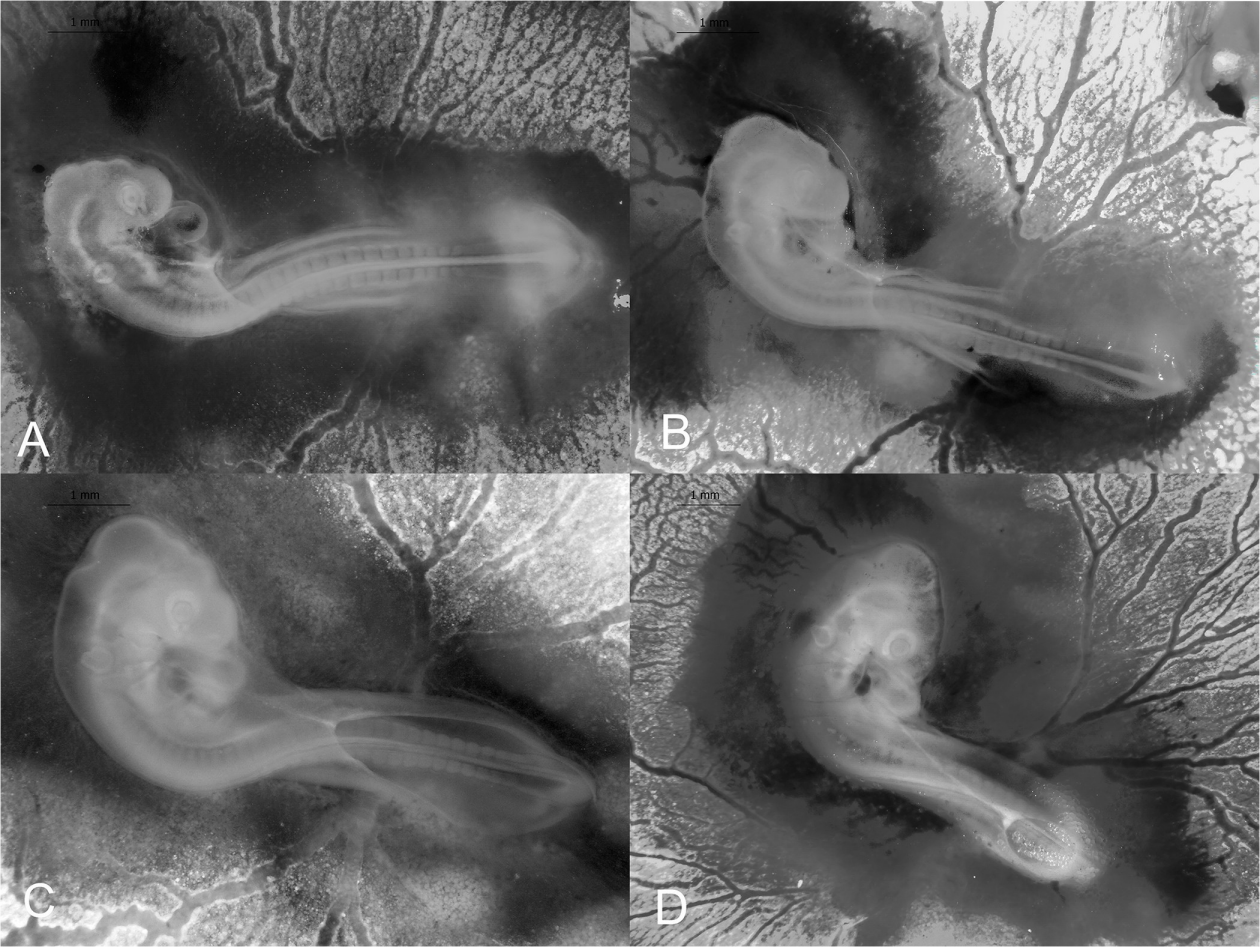
Stages 15–18 (66–84 h) of the California valley quail’s embryological stages of development. (A) Stage 15, 24 somite pairs present. Optic cup and double contour has formed. (B) Stage 16, 26–28 somite pairs present. (C) Stage 17, nasal pits are visible and amnion extends toward trunk. (D) Stage 18, first appearance of wing-bud. Amnion enclosed except for small oval on the trunk.

Twenty-four somite pairs present. Optic cup and double contour has formed. Vitelline veins are much more prominent than previous stages. Third branchial arch is present. The forebrain and hindbrain create an acute angle. Rotation is more accentuated than previous stages. Amnion extends to the somite pairs 7–10. Embryo length: 7.22 ±0.47 mm (*N* = 2; Fig 5A).

#### Stage 16 (72–74 h): 26–28 somite pairs

Twenty-six to twenty-eight somite pairs visible. Cranial flexure extends to somite pairs 14–15. Embryo length: 7.13 ±0.57 mm (*N* = 3; Fig 5B).

#### Stage 17 (74–84 h)

Nasal pits visible. Limb-areas still flat. Cervical flexure is bent more towards the body, but the angle is still >90°. Amnion extends toward trunk. Allantois not yet formed. Embryo length: 6.71 ±0.16 mm (*N* = 5; Fig 5C).

#### Stage 18 (78–84 h)

First appearance of wing-bud. Darkening ridge of cells demarcate the beginning of wing formation. Tail bud has rotated to the right. Amnion enclosed except for small oval on the trunk. Embryo length: 6.85 ±0.91 mm (*N* = 6; Fig 5D).

#### Stage 19 (90 h)

**Fig 6:**
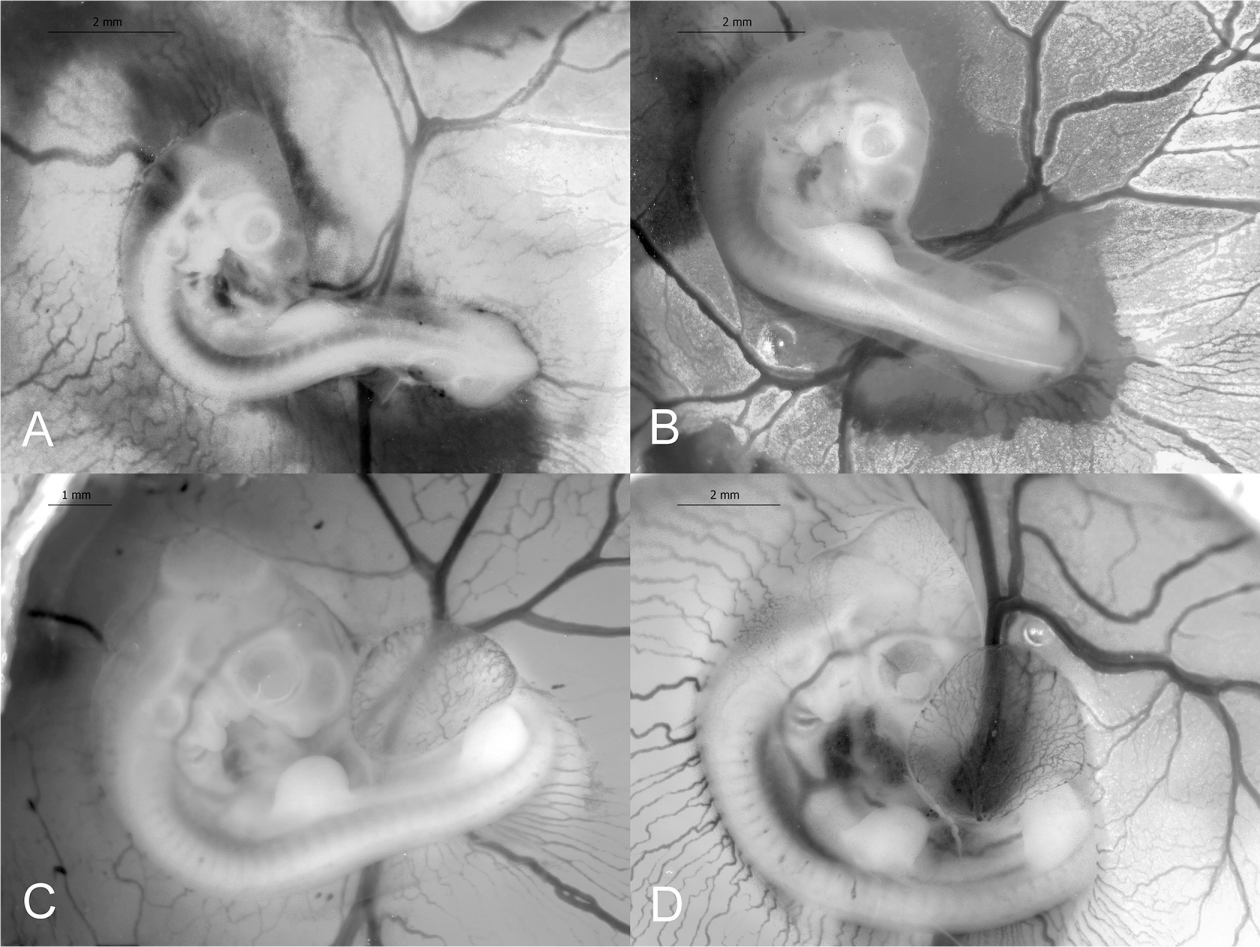
Stages 19–22 (90–120 h) of the California valley quail’s embryological stages of development. (A) Stage 19, limb buds are slightly enlarged, and maxillary process has become distinct. (B) Stage 20, wing-buds are longer than leg-buds. (C) Stage 21, wing- and leg-buds slightly asymmetrical and enlarged. (D) Stage 22, posterior contours of leg- and wing-buds are steeper than anterior contours.

Limb-buds are slightly enlarged. Maxillary process has become distinct. The 2nd visceral arch protrudes slightly. Tail-bud is curved and pointing toward the body. Diencephalon is at wing level. Wing-bud length: 1.12 ±0.10 mm (*N* = 3). Wing-bud width: 0.30 ±0.08 mm (*N* = 3). Embryo length: 7.16 ±0.48 mm (*N* = 2; Fig 6A).

#### Stage 20 (90–96 h)

Wing-buds are longer than leg-buds. Both wing- and leg-buds are longer than wide. Arches I and II are equal in length. The third arch extends in length and becomes prominent. Embryo has rotated towards itself and completed rotation. The embryo’s trunk is in a straight line. Allantois has thinned and extended to head. Eye pigmentation is faint. Wing-bud length: 1.11 ±0.04 mm (*N* = 4). Wing-bud width: 0.28 ±0.03 mm (*N* = 4). Leg-bud length: 0.97 ±0.05 mm (*N* = 4). Leg-bud width: 0.29 ±0.02 mm (*N* = 4). Embryo length: 6.13 ±0.40 mm (*N* = 5; Fig 6B).

#### Stage 21 (102–108 h)

Both wing- and leg-buds slightly asymmetrical and enlarged. Posterior contours of wing- and leg-buds are close to 90°. Anterior contour are ~45°. Maxillary process distinct and exceeds the mandibular process. The 4th cleft is present as a slit. Allantois has become vesicular. Eye pigmentation still faint. Wing-bud length: 1.13 ±0.03 (*N* = 7). Wing-bud width: 0.49 ±0.05 mm (*N* = 7). Leg-bud length: 1.05 ±0.05 mm (*N* = 5). Leg-bud width: 0.50 ±0.10 mm (*N* = 5). Embryo length: 6.79 ±0.10 mm (*N* = 3; Fig 6C).

#### Stage 22 (114–120 h)

Posterior contours of leg- and wing-buds are steeper than anterior contours. Circumference of eye is darkened with pigment. Wing-bud length: 1.07 ±0.03 mm (*N* = 3). Wing-bud width: 0.88 ±0.05 mm (*N* = 3). Leg-bud length: 1.23 ±0.06 mm (*N* = 3). Leg-bud width: 0.86 ±0.27 mm (*N* = 3). Embryo length: 7.10 ±0.15 mm (*N* = 3; Fig 6D).

#### Stage 23 (114–120 h)

**Fig 7:**
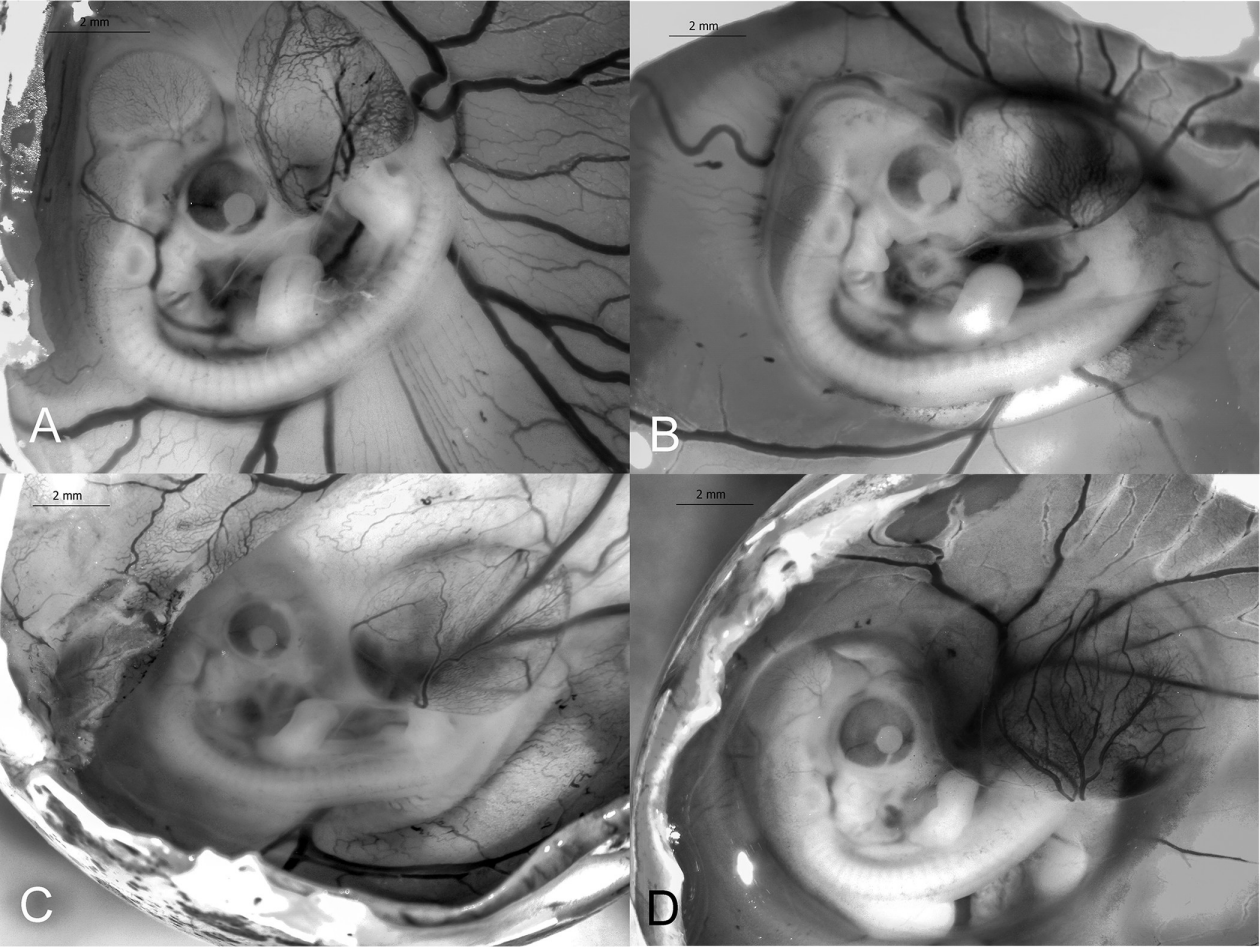
Stages 23–26 (114–144 h) of the California valley quail’s embryological stages of development. (A) Stage 23, anterior and posterior contours of limb-buds run parallel. (B) Stage 24, limbs have lengthened and are longer than wide. (C) Stage 25, all visceral protuberances have fused together and flattened. (D) Stage 26, wings bent at elbow and knee joints distinct.

Both wing- and leg-buds equal in length. Anterior and posterior contours run parallel. Both wing- and leg-buds approximately equal in length as width. Maxillary process lengthened. The dorsal contour of the entire body from the hindbrain to tail is a curved line. Wing-bud length: 1.18 ±0.02 mm (*N* = 3). Wing-bud width: 0.98 ±0.06 mm (*N* = 3). Leg-bud length: 1.18 ±0.03 mm (*N* = 2). Leg-bud width: 0.71 ±0.17 mm (*N* = 2). Embryo length: 7.54 ±0.46 mm (*N* = 3; Fig 7A).

#### Stage 24 (5 d)

Wing- and leg-buds are distinctly longer than previous stage. Limbs have lengthened and are longer than wide. Toe-plate barely recognizable. Wing and toes are not yet demarcated. Choroid fissure present. Wing-bud length: 1.28 ±0.17 mm (*N* = 2). Wing-bud width: 1.00 ±0.08 mm (*N* = 2; Fig 7B).

#### Stage 25 (5.5–6 d)

Wing-buds are slightly longer than previous stage. All visceral protuberances have fused together and flattened. Maxillary process has reached nasal groove. Wing-bud length: 2.12 ±0.26 mm (*N* = 3). Wing-bud width: 0.94 ±0.11 mm (*N* = 3; Fig 7C).

#### Stage 26 (5.5–6 d)

Wings bent at elbow and knee joints distinct. Contour of digital plate is angular in the first digit area. Digit and toe plate distinct. Wing-bud length: 2.22 ±0.19 mm (*N* = 2). Leg-bud width: 0.99 ±0.05 mm (*N* = 2; Fig 7D).

#### Stage 27 (6–6.5 d)

**Fig 8:**
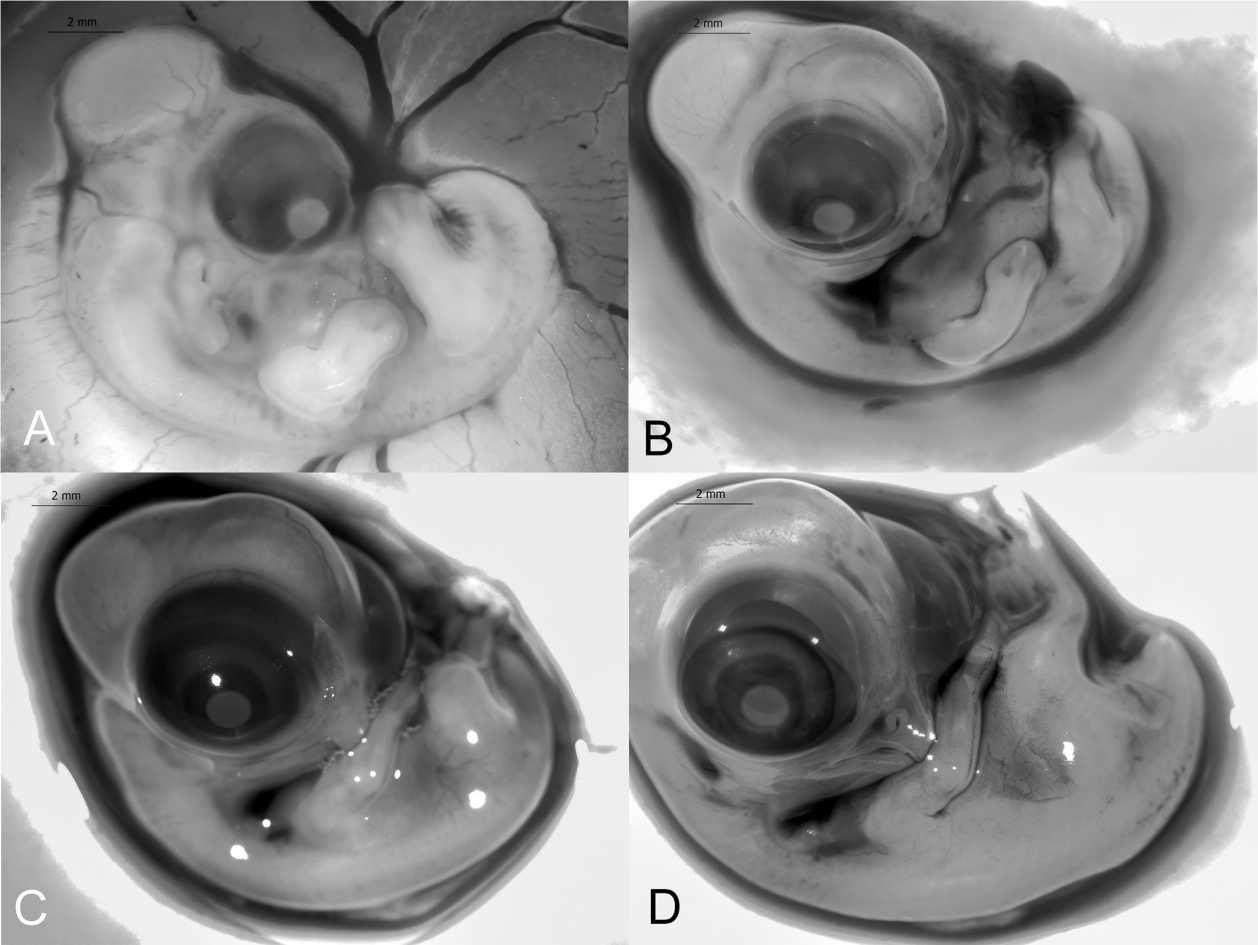
Stages 27–30 (6–11 d) of the California valley quail’s embryological stages of development. (A) Stage 27, digits are concave, and 4 toes are conspicuous. (B) Stage 28, wing tip and digital plate are more angular and webbing between digits is more concave. (C) Stage 29, elbow joint is more pronounced. (D) Stage 30, egg tooth is present.

Digits are concave; 4 toes conspicuous. Beak is barely recognizable. Wing-bud length: 2.64 ±0.19 mm (*N* = 4). Leg-bud length: 3.64 ±0.17 mm (*N* = 5; Fig 8A).

#### Stage 28 (7–8d)

Wing tip and digital plate are more angular. Webbing between digits is more concave and third toe has lengthened. Beak outgrowth is recognizable in embryo profile. Nictitating membrane begins to cover outer edge of eye. Wing-bud length: 3.46 ±0.12 mm (*N* = 7). Wing-bud width: 1.20 ±0.09 mm (*N* = 7). Third toe length: 1.08 ±0.03 mm (*N* = 2). Embryo length: 13.95 ±1.11 mm (*N* = 3; Fig 8B).

#### Stage 29 (8–9 d)

Elbow joint more pronounced; knee bent at knee-joint. Second digit and third toe have lengthened. Digital plate is more angular in appearance than previous stage. Neck between collar and mandible has lengthened. Beak is distinct and much more prominent than previous stage. No egg tooth present. Beak length: 1.56 ± 0.05 mm (*N =* 4; Fig 8C).

#### Stage 30 (9 d)

The 3 major segments of the wing are defined. Scleral papillae present near choroid fissure. Egg tooth is present. Length of beak (nostril to tip): 1.02 ±0.05 mm (*N* = 6). Third toe length: 2.76 ±0.19 mm (*N* = 9). Embryo length: 7.09 ±0.06 mm (*N* = 3; Fig 8D).

#### Stage 31 (10–11 d)

**Fig 9:**
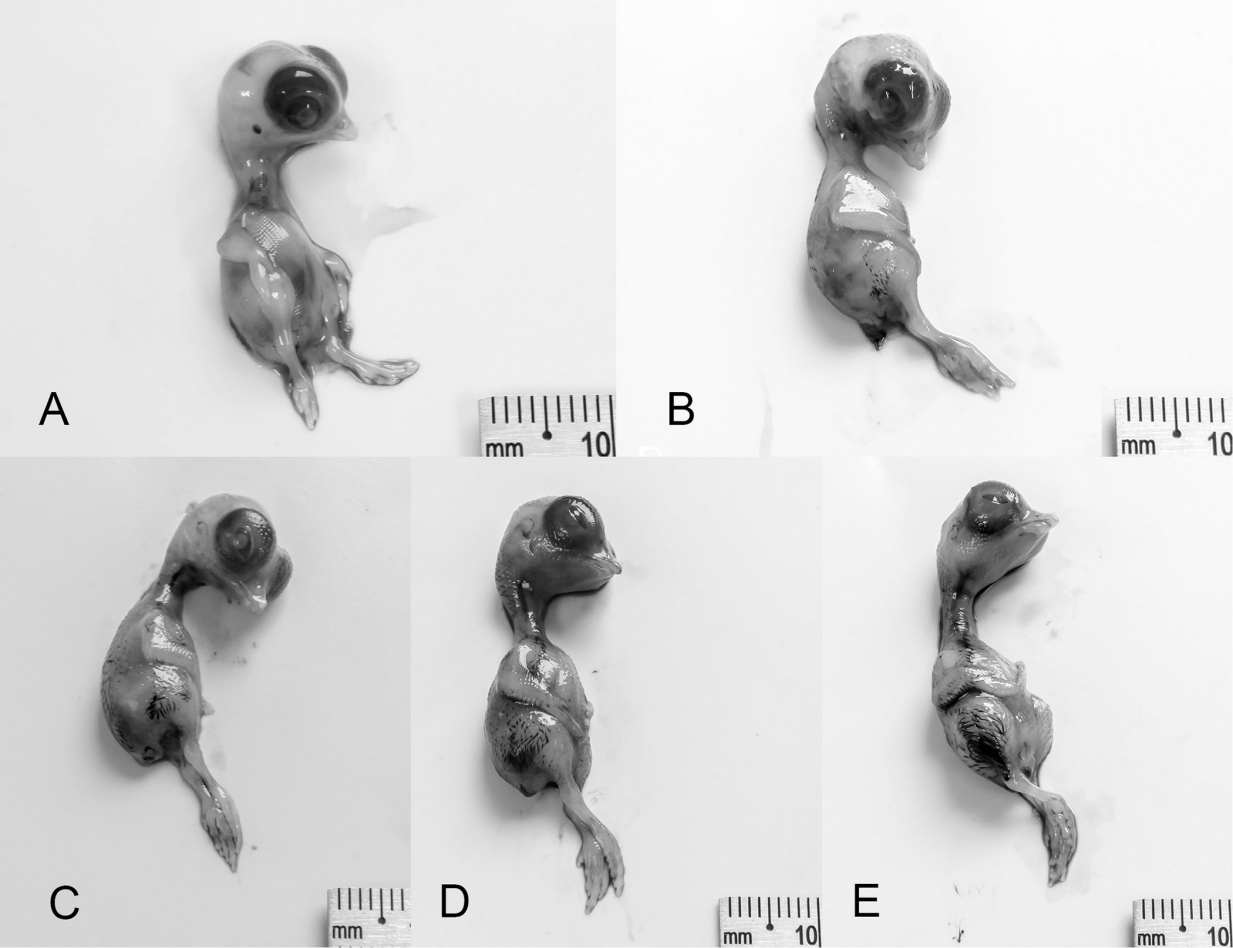
Stages 31–35 (10–14 d) of the California valley quail’s embryological stages of development. (A) Stage 31, primordia of claws. (B) Stage 32, 2 dorsal rows of pigmentation on either side of spine. (C) Stage 33, 4 dorsal rows of pigmentation of either side of spine. (D) Stage 34, brown pigmented feather tips appear along spine. (E) Stage 35, brown pigmentation identified on crown, anterior region of wing and along the thigh.

Primordia of claws. Narrowed gap between mandible and beak. Nostrils form a slit. Circumference of eyelids are in a teardrop shape. Feather-germs present. No pigmentation of feather germs. Third toe length: 3.19 ±0.28 mm (*N* = 7; Fig 9A).

#### Stage 32 (11–12 d)

Neck has lengthened. Eyelids begin to approach corneas. Scleral papillae almost form complete circle. 2 dorsal rows of pigmentation on either side of spine. 1 row of pigmented feathers near thigh. Length of beak (nostril to tip): 1.53 ±0.06 mm (*N* = 7). Third toe length: 4.84 ±0.10 mm (*N* = 7; Fig 9B).

#### Stage 33 (12–13 d)

Four dorsal rows of pigmentation of either side of spine. 4 to 5 rows of pigmented feathers near outer thigh. 2 to 3 rows of feather germs around external auditory pit. Length of beak (nostril to tip): 1.71 ±0.06 mm (*N* = 6). Third toe length: 5.54 ±0.22 mm (*N* = 6; Fig 9C).

#### Stage 34 (13 d)

Pads on plantar surface present. Nailbed more developed. No primordia of scales on feet. Brown pigmented feather tips appear along spine. 2–3 rows of pigmentation on the posterior region of the wing. 12–16 rows of pigmentation anterior region of head. Majority of body covered in feather germs. Length of beak (nostril to tip): 1.82 ±0.05 mm (*N* = 8). Third toe length: 6.37 ±0.09 mm (*N* = 8; Fig 9D).

#### Stage 35 (14 d)

All feathers have lengthened and cover dorsal and lateral sides. Brown pigmentation identified on crown, anterior region of wing and thigh. Length of beak (nostril to tip): 2.03 ±0.07 mm (*N* = 6). Third toe length: 7.25 ±0.16 mm (*N* = 6; Fig 9E).

#### Stage 36 (15 d)

**Fig 10:**
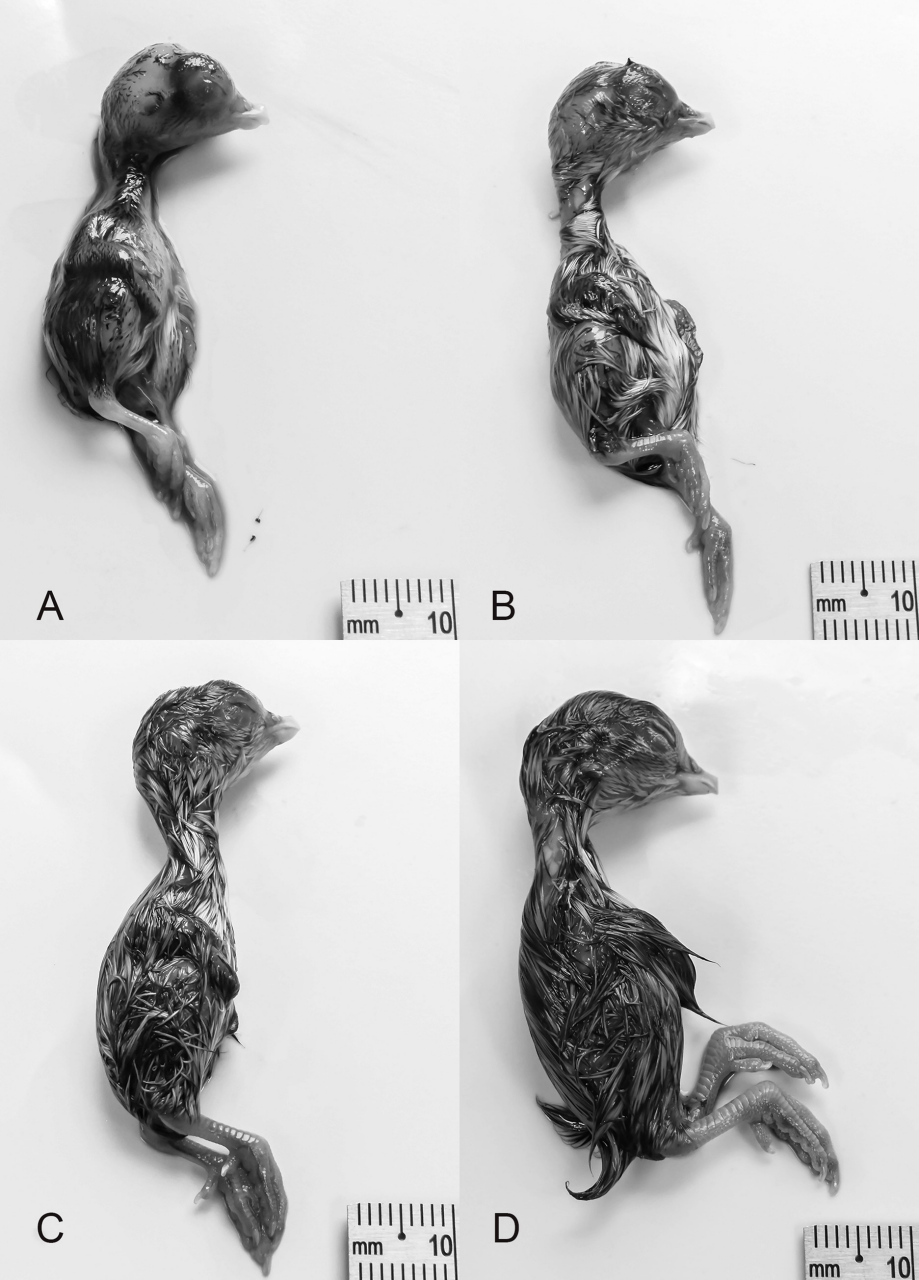
Stages 36–39 (15–18 d) of the California valley quail’s embryological stages of development. (A) Stage 36, primordia of scales on legs. (B) Stage 37, white feathers are present. (C) Stage 38, periderm beginning to peel off the beak. (D) Stage 39, scales begin to overlap.

Primordia of scales on legs. White feathers are present on ventral region of embryo. Length of beak (nostril to tip): 2.24 ±0.03 mm (*N* = 7). Third toe length: 8.23 ±0.15 mm (*N* = 8; Fig 10A).

#### Stage 37 (16 d)

Feathers have lengthened over entire body. Eyelid openings have reduced in size. Length of beak (nostril to tip): 2.50 ±0.07 mm (*N* = 5). Third toe length: 9.71 ±0.10 mm (*N* = 8; Fig 10B).

#### Stage 38 (17 d)

Periderm is thick and beginning to peel off due to the egg tooth’s cornification [19]. Scales begin to overlap on inferior and superior surfaces of the leg. Length of beak (nostril to tip): 2.75 ±0.07 mm (*N* = 8). Third toe length: 10.61 ±0.18 mm (*N* = 8; Fig 10C).

#### Stage 39 (18 d)

Periderm is completely absent. Eyes are almost completely closed. Beak is brown with white egg-tooth visible. Length of beak (nostril to tip): 2.95 ±0.10 mm (*N* = 8). Third toe length: 12.25 ±0.17 mm (*N* = 8; Fig 10D).

#### Stage 40 (19–20 d)

**Fig 11:**
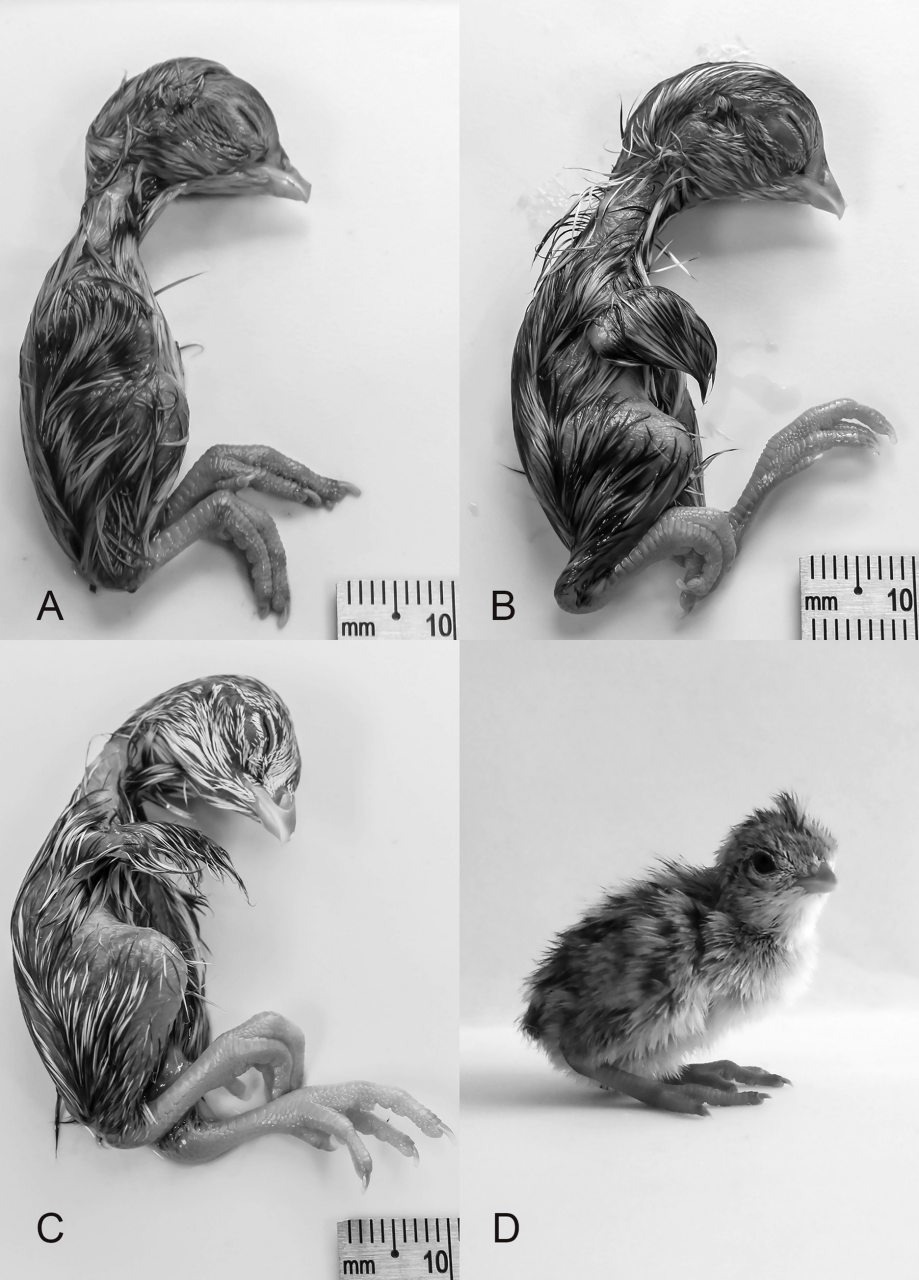
Stages 40–43 (19–23 d) of the California valley quail’s embryological stages of development. (A) Stage 40, beak is more pointed than previous stage. (B) Stage 41, scales overlapping. (C) Stage 42, yolk-sac two-thirds to almost entirely inside body cavity. (D) Stage 43, newly hatched chick.

Beak is more pointed than previous stage. The upper portion of the beak has turned dark brown. Eyelids completely closed. Length of beak (nostril to tip): 3.22 ±0.05 mm (*N* = 25). Third toe length: 13.38 ±0.33 mm (*N* = 26; Fig 11A).

#### Stage 41 (20–21 d): Internally-pipped

Scales overlapping on inferior and superior surfaces of leg. Claws curve sharply toward body. Yolk-sac one-third inside body cavity. Length of beak (nostril to tip): 3.26 ±0.10 mm (*N* = 10). Third toe length: 13.27 ±0.39 mm (*N* = 10; Fig 11B).

#### Stage 42 (20–22 d): Star-pipped

Yolk-sac two-thirds to almost entirely inside body cavity. Chorio-allantoic membrane is sticky in the eggshell. Length of beak (nostril to tip): 3.54 ±0.05 mm (*N* = 13). Third toe length: 13.62 ±0.17 mm (*N* = 13; Fig 11C).

#### Stage 43 (23 d): Hatch

Newly hatched chick. Yolk-sac is completely enclosed inside body cavity. Total beak length: 5.56 ±0.14 mm (*N* = 5; Fig 11D).

## Discussion

**Fig 12:**
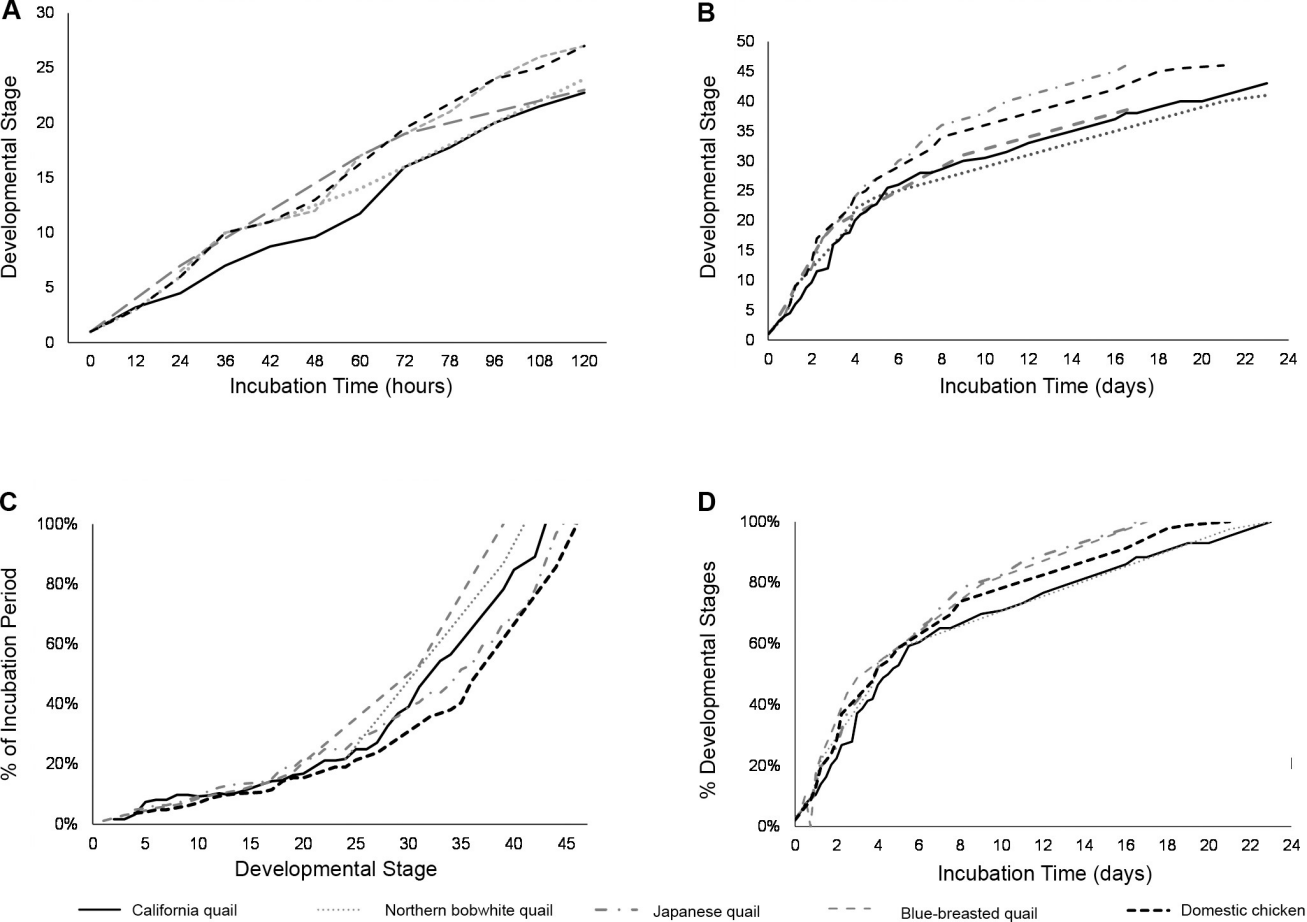
Comparisons of developmental stages of California quail, northern bobwhite quail, Japanese quail, blue-breasted quail, and domestic chicken embryos. (A) Stages for the first 5 days of development. (B) Stages for entire incubation period (≤23 days). (C) Comparison of stages and percentage of total incubation time. (D) Comparison of stages and of percentage of development.

The California valley quail embryo has 43 normal stages of development, which differs from the number of developmental stages from avian models and close galliform relatives. Domestic chickens and Japanese quail embryos have 46 stages of development [6,7], blue-breasted quail have 39 stages [18], and northern bobwhite quail have 41 stages [17]. The chronology of development for the California valley quail differs from other quail in stages 1–24 (1–5 d; Fig 12A), and the morphological and structural development differs from the domestic chicken in stages 25–hatch (6–23 d; Fig 12B). When incubation time is standardized, California valley quail development is similar to other staged galliforms (Fig 12C). When development is standardized, California quail lag behind other staged galliforms (Fig 12D). The observed differences in the number and timing of stages of development emphasize the importance of staging avian species of interest instead of relying on animal models and close relatives for reference. Caution should be exercised when extrapolating data from poultry species like chickens and Japanese quail to other birds due to the multiple generations of selection and domestication >10,000 years ago [20]. This is extremely important in species-specific embryological studies that access critical windows of development [21] and evaluate the impacts of environmental change on avian development [22–25].

The observed differences in the number of stages of development also emphasize the need for establishing standard observation frequencies when staging avian embryos. We documented embryos in increments of ≤6 hours, similar to studies that staged the domestic chicken [7], Japanese quail [6], and blue-breasted quail [18]. However, northern bobwhite quail were only observed every 24 hours when staged [17], resulting in the possibility of missed stages. Future embryological studies, including a more thorough investigation of northern bobwhite development, would benefit from examining embryos in increments of ≤6 hours to document all morphological stages more accurately.

Although we compared developmental timing and the number of stages between galliforms, avian development is influenced by temperature and humidity during incubation, which vary in embryological staging studies. Incubation temperatures for domestic chickens where 39.4°C, 37.5°C, and 38°C, with no humidity reported [7], Japanese quail were incubated at 38°C with 70% RH [6], and blue-breasted quail at 37.7°C with 70% RH [18]. Northern bobwhites were incubated at 37.5°C with 60% RH [24], and a 31.7°C wet bulb, or ~66% RH [17]. We incubated California valley quail at 37.5°C with 60% RH; the same as northern bobwhites [24]. It is unknown how these slight differences influence the comparison of stages since incubation temperature and humidity are species dependent.

Normal stages of development are required to determine the impacts of stressors and teratogens on embryo development [21]. Factors such as heat stress from drought [21,22] and hazardous pesticides have been shown to drastically affect the development of other quail species [23–25], and have major consequences for population sustainability [26,27]. California valley quail are considered highly vulnerable to climate change due to increased fire risk, and spring heat waves disrupting reproduction [28]. The stages produced in this study could help determine the impact of high temperatures on developing California valley quail embryos.

One often overlooked factor in staging studies is the condition of the eggs from hatchery to laboratory. During this study, several eggs failed to develop and hatch. Although this is normal during studies with eggs, varying shipping conditions could have influenced hatchability [7,31]. Shipping, and receiving temperatures can exceed physiological zero [29], and reach threatening or lethal levels [22–26]. This may result in altered development [21,23,24], affecting the recorded embryological stages. Our eggs were packaged in foam within cardboard boxes, shipped during the spring within 24 hours of collection, and quickly processed on the receiving end. Since spring temperatures were mild and the packaging offered some thermal buffering, we don’t suspect that shipping conditions greatly influenced our results. However, future research directed at documenting normal stages of development would benefit from controlling and recording hatchery, shipping, and receiving conditions. We hope that this series of normal staging and recommendations will be useful as a laboratory aid for future embryological investigations.

## Materials and methods

All research was approved by the Texas A&M University-Commerce’s Institutional Animal Care and Use Committee; Animal Use Protocol P21-026.

### Egg acquisition

Fertilized California valley quail eggs (*N* = 390) were collected and shipped, within 24 hours, from Minnesota (Stromberg’s Chick and Game Birds Unlimited, Hackensack, MN) to The Quail Research Laboratory at Texas A&M University–Commerce. In 2020, 3 shipments (*N* = 290 eggs) were delivered over 12 weeks, April–June. In 2021, 1 shipment (*N* = 100 eggs) was delivered in May. Staggered shipments in 2020 provided sufficient time to incubate eggs in each shipment. Upon arrival, eggs were inspected for fine-line cracks and broken shells by candling. Eggs with cracks or broken shells were discarded. After inspection, eggs were weighed to the nearest 0.01 g using a portable balance (Scout SPX222, Ohaus, Parsippany, New Jersey), labeled with a permanent marker, and incubated at 37.5°C with ~60% RH in a cabinet incubator (1502 Digital Sportsman, GQF, Savannah, GA, USA). Eggs were automatically turned every hour to mimic natural incubation [18] and opened daily, during specific time periods, to examine embryos and record key morphological characteristics and structures.

### Staging embryonic development

**Fig 13:**
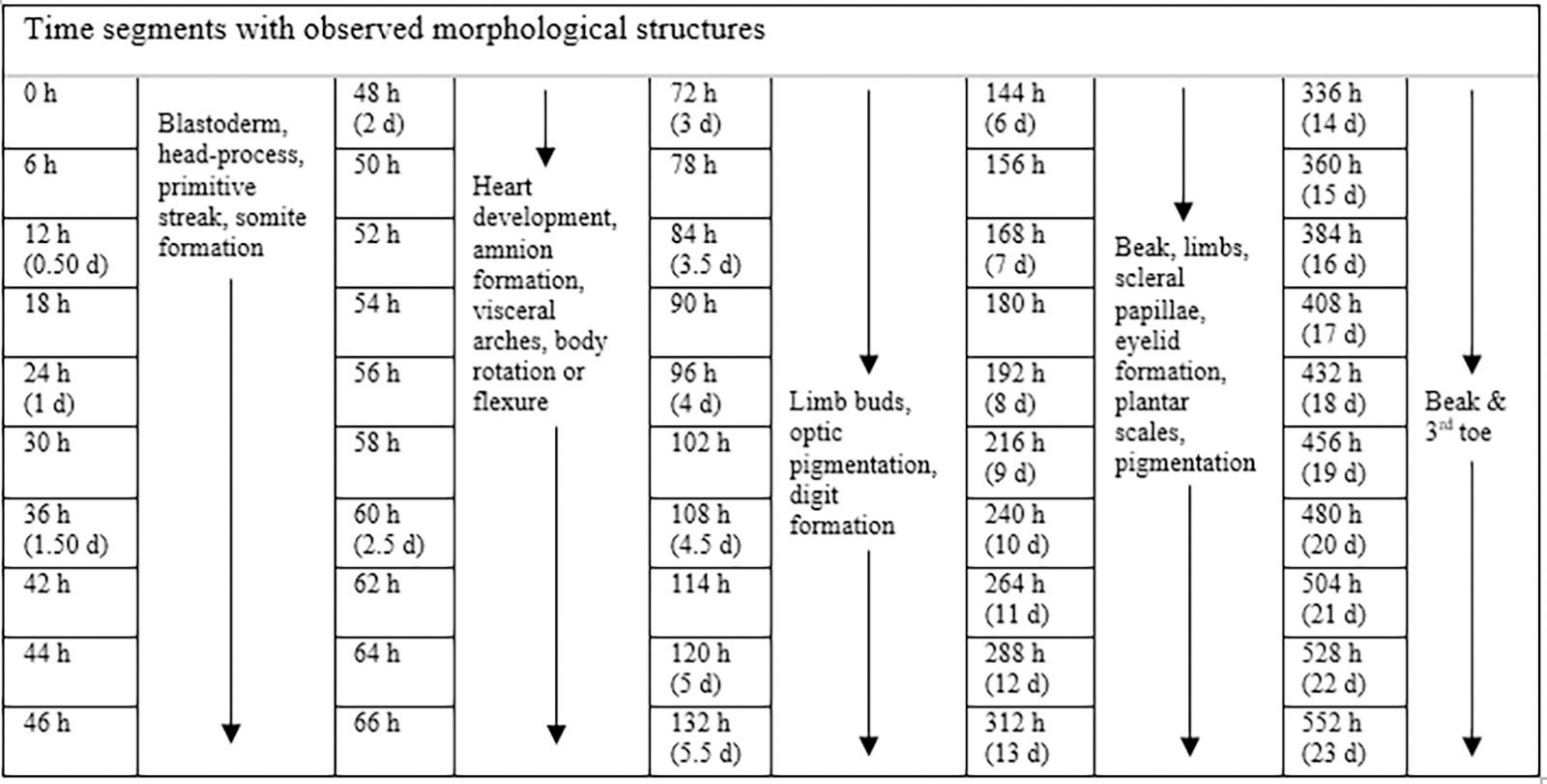
Time segments used for opening California valley quail eggs with observed key morphological structures for developing normal stages of development.

To stage California valley quail embryo during incubation, key morphological characteristics and structures were documented for 50 time segments (Fig 13). For each time segment, 5 eggs were randomly selected, placed on an 11-mm rubber stage under a dissecting microscope (M80/IC90E, Leica Camera, AG, Wetzlar, Germany), and opened by carefully removing 2–4 mm of the egg’s blunt end with a 25-gauge needle and fine-pointed forceps. Once opened, blue methylene (0.1–0.25 ml) was injected directly under the embryo using a 25-gauge syringe to enhance visibility and increase contrast.

Measurements recorded from opened eggs included blastoderm diameter, anterior angle of nostril to beak tip, and lengths of wing, tarsus, third toe, total foot, total beak, and embryo, when extended [7,14,30]. Limb-bud length was measured proximal to distal and width was measured anterior to posterior [7]. The first pair of somites of the series was not clearly defined; therefore, not counted [7,18]. Photographs were taken of all opened eggs and developing embryos. Visibility of photographed embryo was improved by adjusting contrast, exposure, highlight, shadows, whites, blacks, and clarity using image editing software (Photoshop Lightroom 19.1.4, Adobe, Inc., Mountain View, CA, USA). Editing adjustments varied depending on visibility of relevant structures. Key morphological structures were identified, measured, and compared to developmental studies of other galliforms [6,7,17,18]. This study is different from other descriptions of normal embryonic staging because embryos were photographed *in ovo* instead of analyzing preserved or fixed embryos. Once the first year of research was concluded, all data were analyzed, and stages were determined according to structural developments and chronological findings. Any missing or poorly defined stages were prioritized for year 2. Embryos incubated ≥10 days were euthanized in a closed chamber with 90% CO_2_ for ≥20 minutes [31–33]. Embryos incubated >20 days were handled in accordance with Texas A&M University–Commerce IACUC protocol P21-026.

All statistical analyses were performed using Microsoft Excel (Version 2011, Redmond, WA). All measurements are reported as mean ±SD. Statistical decisions were made at an alpha <0.05.

## Supporting information

S1 Data (XLSX)
